# Identification of MyoD-Responsive Transcripts Reveals a Novel Long Non-coding RNA (lncRNA-AK143003) that Negatively Regulates Myoblast Differentiation

**DOI:** 10.1038/s41598-017-03071-7

**Published:** 2017-06-06

**Authors:** Yiwen Guo, Jingnan Wang, Mingfei Zhu, Rui Zeng, Zaiyan Xu, Guoliang Li, Bo Zuo

**Affiliations:** 1 Key Laboratory of Swine Genetics and Breeding of Ministry of Agriculture & Key Laboratory of Agriculture Animal Genetics, Breeding and Reproduction of Ministry of Education, College of Animal Science, Huazhong Agricultural University, Wuhan, 430070 Hubei P.R. China; 2 The Cooperative Innovation Center for Sustainable Pig Production, Wuhan, 430070 China; 3 National Key Laboratory of Crop Genetic Improvement, Agricultural Bioinformatics Key Laboratory of Hubei Province, College of Informatics, Huazhong Agricultural University, Wuhan, 430070 Hubei P.R. China

**Keywords:** Gene expression, Gene regulation

## Abstract

Myogenic differentiation factor (MyoD) is a master transcription factor in muscle development and differentiation. Although several long non-coding RNAs (lncRNAs) linked to MyoD have been found to influence muscle development, the functions of many lncRNAs have not been explored. Here we utilized lncRNA and mRNA microarray analysis to identify potential lncRNAs regulated by MyoD in muscle cells. A total of 997 differentially expressed lncRNAs (335 up-regulated and 662 down-regulated) and 1,817 differentially expressed mRNAs (148 up-regulated and 1,669 down-regulated) were identified after *MyoD* knockdown in C2C12 cells. Functional predictions suggested that most lncRNAs are involved in the biological pathways related to muscle differentiation and cell cycle with co-expressed genes. To gain further insight into the MyoD-mediated lncRNA expression in muscle differentiation, tissue expression profiles and *MyoD* overexpression were performed, and we found one of the candidate lncRNAs-AK143003 was significantly regulated by MyoD. Further analyses showed its noncoding ability and cytoplasmic localisation. Silencing of AK143003 stimulated the accumulation of myogenic marker genes, whereas AK143003 overexpression led to their decreased synthesis. This study identified a multitude of MyoD-mediated lncRNAs for further investigation and identified a novel lncRNA, lnc-AK143003, which plays a role in controlling muscle differentiation.

## Introduction

Muscle cell differentiation is mainly regulated by muscle specific transcription factors. Thus far, the muscle regulatory factors (MRFs) as a paradigm of structurally-related basic-helix-loop-helix (bHLH) transcription factors were identified to orchestrate the activation of muscle-specific transcription^1^. The members of MRF genes expression displays subtle correlativity in myogenesis and is dependent on myogenic differentiation factor (MyoD). In the proliferating cells, myogenic factor (Myf5) and MyoD were found to be co-expressed and bound to the same genes sites, whereas MyoD could more robustly recruit Pol II to bind the promoter of downstream genes^2^, including MyoG. As a myogenic regulator, MyoD possessed the ability of inducing cell cycle withdrawal to trigger differentiation^3–5^. Ablation of *MyoD* expression in C2C12 cells results in the inability to form myotubes, whereas remedial rescue can restore the ability to generate myotubes^6^. *MyoD* knockout mice displayed a dramatic loss of myosin heavy chain (MyHC) protein, a hallmark of fibre formation^6^, furthermore, these mice showed disturbed neuromuscular junctions, an essential component for efficient motor control of skeletal muscles^7^. Moreover, MyoD could directly or indirectly contribute to switching other types of cells—such as smooth muscle cells, adipocytes, neuroblastoma, hepatoma, and baby hamster kidney cells—into muscle cells^1, 8^.

Current advances in high-throughput sequencing technologies have increased the knowledge of noncoding RNAs. One class of them, called long non-coding RNA (lncRNA), was characterized by being over 200 bp in length, often polyadenylated, exhibiting poor expression and conservation^9–12^. It was previously deemed nonfunctional junk until several studies revealed that they had diverse biological functions. To date, most studies on lncRNAs have focused mainly on developmental processes^13–15^, post-embryonic pluripotent stem cells differentiation^16–18^, cell cycle signal mediated cell apoptosis and cancer metastasis^19, 20^. Notably, most studies indicated lncRNAs play important roles in a certain tissue- and species- specific expression^11^, but some multifunctional lncRNAs have displayed broadly and conservative expression profiles^21, 22^. *H19*, the earliest discovered lncRNA, is ubiquitous expression. It could recruit histone modification markers to inhibit the transcription of imprinted genes during embryo growth, and it functions to modulate microRNAs involved in skeletal muscle differentiation and regeneration^23–25^. Moreover, *H19* can contribute to tumorigenesis^26^, endometrial development^27^, and renal development^28^.

Recent studies have investigated muscle-derived lncRNAs using myoblast cell lines and animal models. Two lncRNAs, *Linc-YY1* and *Dum*, were shown to exert their function under the control of MyoD; both were located in the cell nucleus and exhibited in a parallel expression of *MyoD* for promoting myoblast differentiation and muscle regeneration^29, 30^. MyoD could directly bind *Linc-YY1* for recruiting the transcriptional factor YY1 to dissociate/bind target promoters to modulate muscle gene activity^30^. *Dum* was induced by MyoD to form intrachromosomal looping with neighboring gene and to silence the expression of Dppa2, thereby delaying differentiation^29^. Another MyoD-dependent lncRNA (*lncMyoD*), which was generated from the upstream region of *MyoD* transcriptional initiation site, was activated by MyoD to suppress the expression of proliferation genes, causing cell cycle exit and promoting terminal differentiation^31^. Despite recent progress in understanding the roles of lncRNAs in the regulation of muscle differentiation, the functions of many MyoD-mediated transcripts remained elusive.

In this study, we used a high-throughput approach combined with available databases to systematically screen MyoD-mediated lncRNAs by microarray analysis. To identify novel lncRNAs in myogenesis, bioinformatic and tissue expression profile analyses were employed to identify candidate lncRNAs that had potentially function in controlling the differentiation of C2C12 cells. One novel lncRNA, lncRNA-AK143003, was found to have important role in regulating the expression of the target muscle marker genes, MyoG and MyHC. Our study provided abundant information and reference value for lncRNAs modulation in muscle differentiation.

## Results

### Identification of differentially expressed lncRNAs and mRNAs regulated by MyoD in C2C12 cells

**Figure 1:**
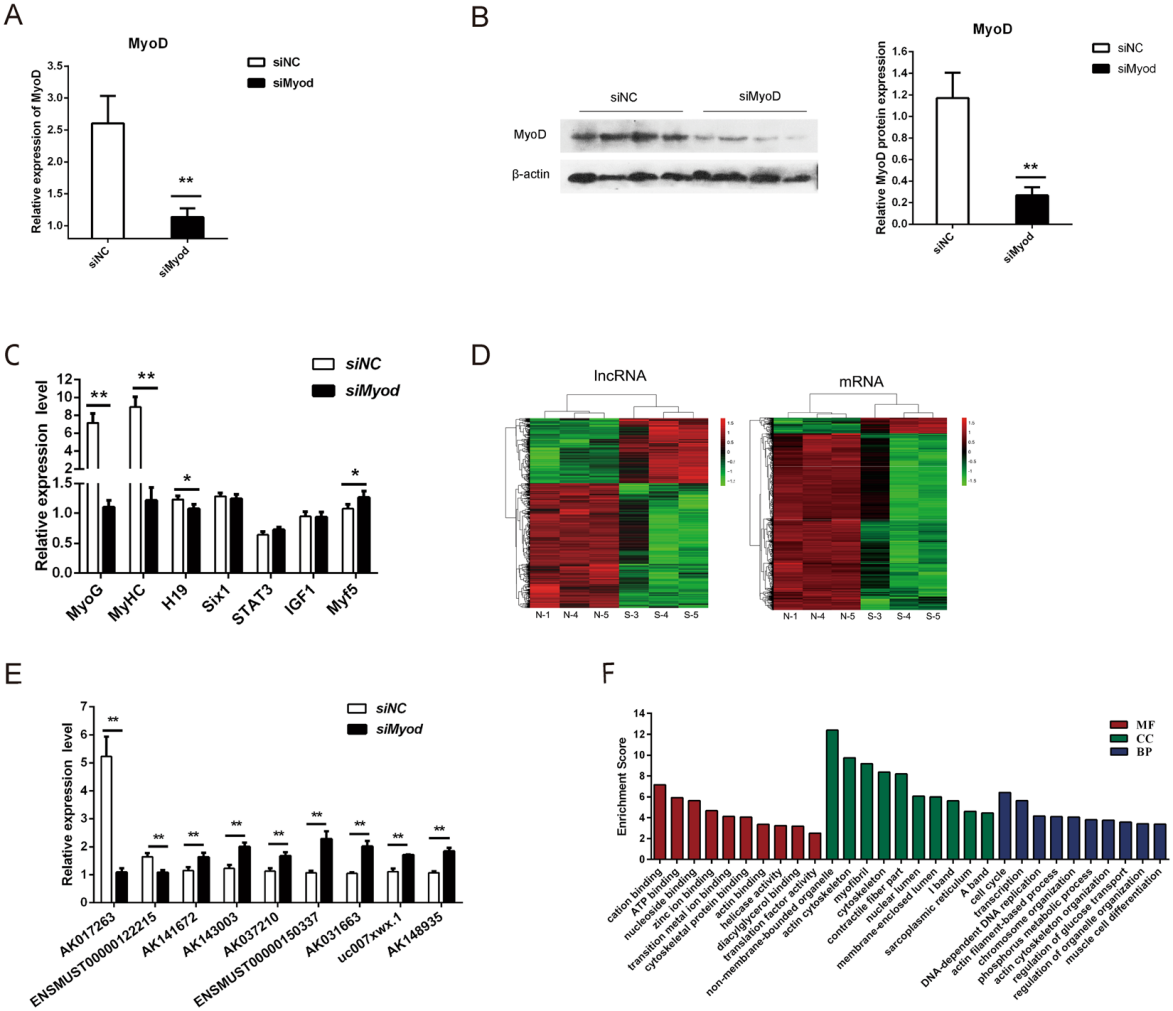
Analysis of differential gene expression profile by *MyoD* interference. (**A** and **B**) Knockdown of *MyoD* by siRNA oligos in differentiated C2C12 cells for 48 h (**C**) The mRNA level of *MyoD* downregulated or upregulated genes expression after *MyoD* silencing. (**D**) Hierarchical clustering dendrogram depicted the relationships among the dysregulated lncRNA expression patterns of samples (fold change ≥1.5; *p* < 0.05) at 48 h differentiation after siMyoD or control oligo treatment. The colors corresponded to a normalized expression value of each transcript (red: high relative expression; green: low relative expression). (**E**) The differential expression of MyoD-mediated lncRNAs was validated by quantitative real-time PCR. All the qPCR data were normalized to *β-actin* and presented as mean ± S.D. (n = 3). *p < 0.05, **p < 0.01. (**F**) The GO annotations of the co-expression genes correlated to lncRNAs. The top 10 enriched GO term of dysregulated mRNAs are shown for biological process (BP), cellular component (CC) and molecular function (MF) according to the values in the fold enrichment.

In an attempt to shed lights on the functional lncRNAs regulated by MyoD during myogenesis, a custom designed microarray platform was used to analyze the expression profiles of lncRNAs and mRNAs after *MyoD* knockdown. Small interfering RNA (siRNA) were utilised to suppress MyoD protein and mRNA expression by more than 60% in differentiated cells for 48 hours (Fig. 1A,B). Knockdown of *MyoD* caused an eight-fold decrease in expression of the downstream marker genes, *MyoG* and *MyHC* (Fig. 1C). And the expression of known downstream target, lncRNA-*H19*, was also significant declined^32^. In contrast, no significant changes were found in other upstream genes or those not regulated by *MyoD*, with the exception of *Myf5*, which was shown to compensate for the function of *MyoD* at the early stage of differentiation (Fig. 1C).

From the expression profiles, we found that 29,901 probes targeted lncRNA, while 20,992 probes targeted mRNA. After data normalization, the box and scatter plots indicated the uniformity and repeatability (Supplementary Fig. 1A,B). A total of 335 lncRNAs and 148 mRNAs were significantly up-regulated in the treatment group compared with the control group (>1.5 fold, *p* < 0.05) (Supplementary Table 1), whereas 642 lncRNAs and 1,669 mRNAs were significantly down-regulated (>1.5 fold, *p* < 0.05) (Supplementary Table 2). Hierarchical clustering of the differentially expressed lncRNAs and mRNAs was shown in Fig. 1D. The classification and distribution of lncRNAs in the genome were shown in Supplementary Fig. 1C and D. Most lncRNAs were located in the intergenic and exon regions of coding gene, and their genome distribution varied widely from the group of mRNAs.

To validate the microarray analysis results, we used real-time quantitative PCR (qPCR) to examine nine randomly selected lncRNAs (Fig. 1E). Consistent with the results of the microarray analysis, qPCR showed that expressions of AK143003, ENSMUST00000150337, AK141672, AK037210, AK031663, uc007xwx.1, and AK148935 were significantly increased (*p* < 0.01) after MyoD repression, while AK017263 and ENSMUST00000122215 were significantly decreased (*p* < 0.01).

### LncRNA and mRNA co-expression profiles and prediction of lncRNA functions

A coding-non-coding gene co-expression (CNC) network was constructed based on Pearson correlation analysis. For each differentially expressed lncRNA, we yielded hundreds of co-expressed mRNAs. By selecting lncRNAs and mRNAs with Pearson’s correlation coefficients higher than 0.99 (*p*-value < 0.01) and combining the differentially expressed mRNAs in the microarray, we identified 1,396 mRNAs in the network. This group displayed 77% accordance with all dysregulated mRNAs, demonstrating the reliability of our chip.

Gene ontology (GO) and Kyoto Encyclopedia of Genes and Genomes (KEGG) database pathway analyses were carried out with respect to the co-expressed mRNAs to predict the function of the differentially expressed lncRNAs. We found the majority of genes were involved in muscle formation and basic biology processes. For the biological processes, the co-expressed genes were enriched in cell cycle, transcription, actin filament-based processes and muscle cell differentiation (Fig. 1F). In terms of the cellular components, the dysregulated targets were mostly enriched in non-membrane-bounded organelle, actin cytoskeleton, myofibril, cytoskeleton and contractile fiber part. Moreover, the results indicated significant enrichment in molecular functions including cation binding, ATP binding, nucleoside binding and actin binding. The pathway analysis revealed 37 enrichment-related pathways including “cell cycle”, “chronic myeloid leukemia” and “tight junction”. The ten most representative processes are listed in Supplementary Fig. 1E.

### Tissue-specific expression analysis of some differentially expressed lncRNAs

In order to identify novel myogenic lncRNAs, we excluded lncRNAs with low expression. After a series of screenings, we obtained 37 intergenic lncRNAs with high expression. To gain further insight into the possible function of the candidate lncRNAs, we utilised qPCR to identify the distribution pattern in mouse tissues. Tissue expression pattern analysis showed a diversified expression pattern (Supplementary Fig. 2). Notably, nine lncRNAs displayed muscle-specific or high expression patterns, of which MM9LINCRNAEXON11961− was highly expressed in the leg muscle and *longissimus dorsi*. AK031663, ENSMUST00000150337, AK017263, and ENSMUST00000154720 were enriched in both fat and muscle tissues. AK141672 showed predominant expression in the heart. Three transcripts (AK160312, AK163925, and ENSMUST00000104935) were strikingly enriched in the tongue. Furthermore, a subgroup of lncRNAs (AV570737, UC008MAU.1, ENSMUST00000053965, ENSMUST00000118172, ENSMUST00000118351, ENSMUST00000168516, MM9LINCRNAEXON11909, and AK118572) showed specific expression in testis tissue. Previous studies indicated that MyoD could regulate neurogenesis and suppress neuroma^33, 34^, suggesting that the lncRNAs mediated by MyoD play an important role in neuro cells. Interestingly, we found enriched expression of AK139402, AK012506, AK048117, and AK148935 in the brain. Seven transcripts (MM9LINCRNAEXON11972−, AK017535, AK052777, MM9LINCRNAEXON12093+, MM9LINCRNAEXON11957−, MM9LINCRNAEXON11969−, and uc007xwx.1) displayed remarkable expression in detoxifying organs. Finally, all of the remaining transcripts exhibited ubiquitous expression.

### Identification of lncRNAs tightly induced by MyoD

**Figure 2:**
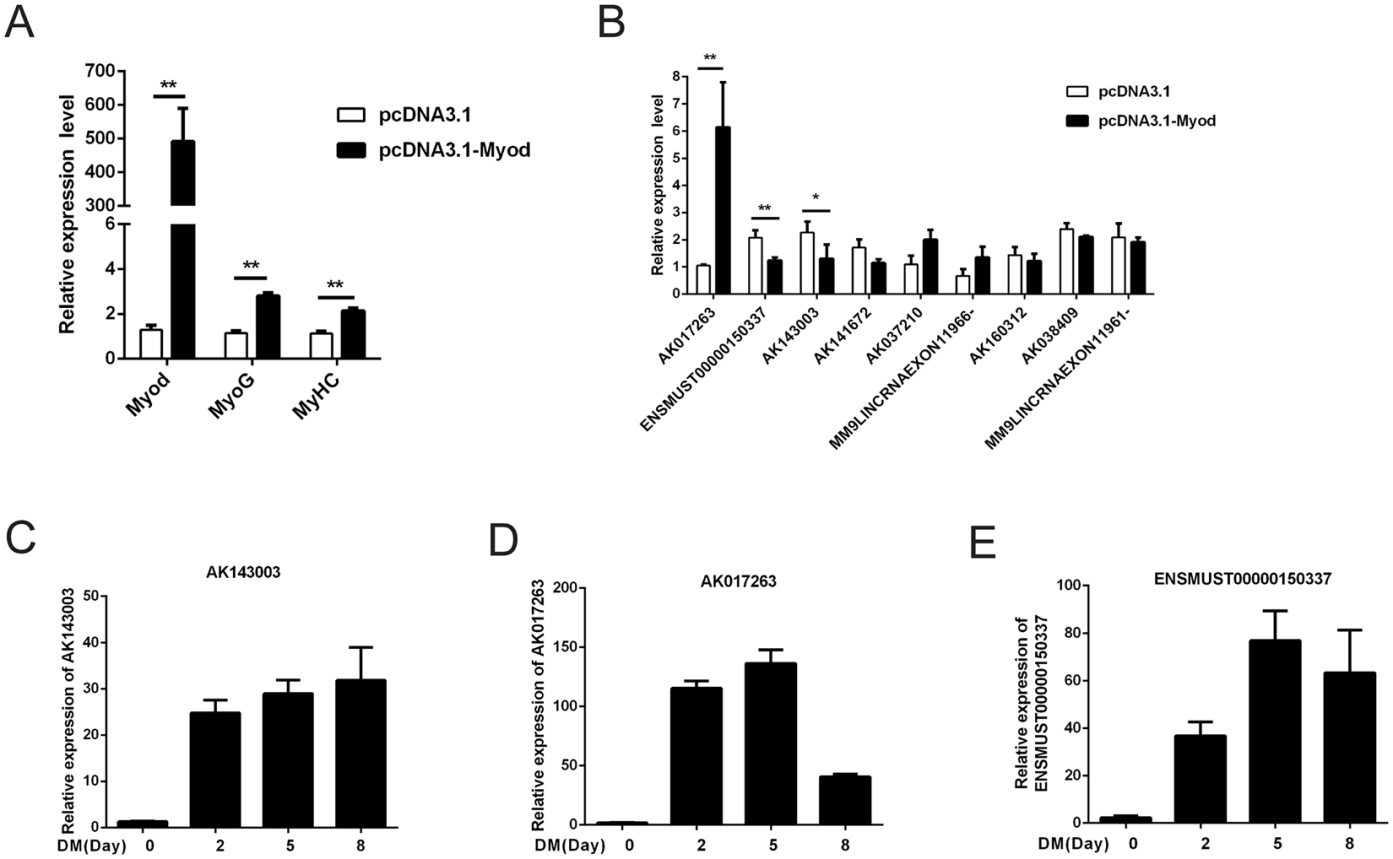
Expression profile of candidate lncRNAs. (**A**) Overexpression of *MyoD* caused the increased mRNA level of *MyoG* and *MyHC* gene. (**B**) The differential expression of lncRNAs from A. (**C**–**E**) The expression of AK017263, ENSMUST00000150337 and AK143003 were monitored by qPCR during the differentiation. All the Q-PCR data were normalized to *β-actin* and presented as mean ± S.D. (n = 3). *p < 0.05, **p < 0.01.

To further investigate whether the identified lncRNAs were positively regulated by the *MyoD* gene, *MyoD* eukaryotic expression vector was used to transfect C2C12 cells. As expected, overexpression of *MyoD* elevated the expression level of the downstream genes *MyoG* and *MyHC* (Fig. 2A). On the other hand, we selected 9 lncRNAs enriched in muscle or all tissues and found the expression of three lncRNAs (AK017263, ENSMUST00000150337, and AK143003) were changed significantly, in accordance with *MyoD* interference (Fig. 2B).

As MyoD is a well-known transcription factor involved in controlling myogenic processes, these results suggested that the three candidate lncRNAs may play an important role in myogenesis. To test this hypothesis, we explored the time course of expression during myoblast differentiation. All three lncRNAs were promptly activated at the early stage of differentiation. However, AK143003 gradually increased until terminal differentiation (Fig. 2C), while AK017263 and ENSMUST00000150337 decreased slightly during differentiation (Fig. 2D and E). It is suggesting that AK017263 and ENSMUST00000150337 are pro-myogenic genes in myogenesis, whereas, AK143003 may play an important role in differentiation and myotube formation.

### Characterisation of AK143003 sequence and subcellular localisation

**Figure 3:**
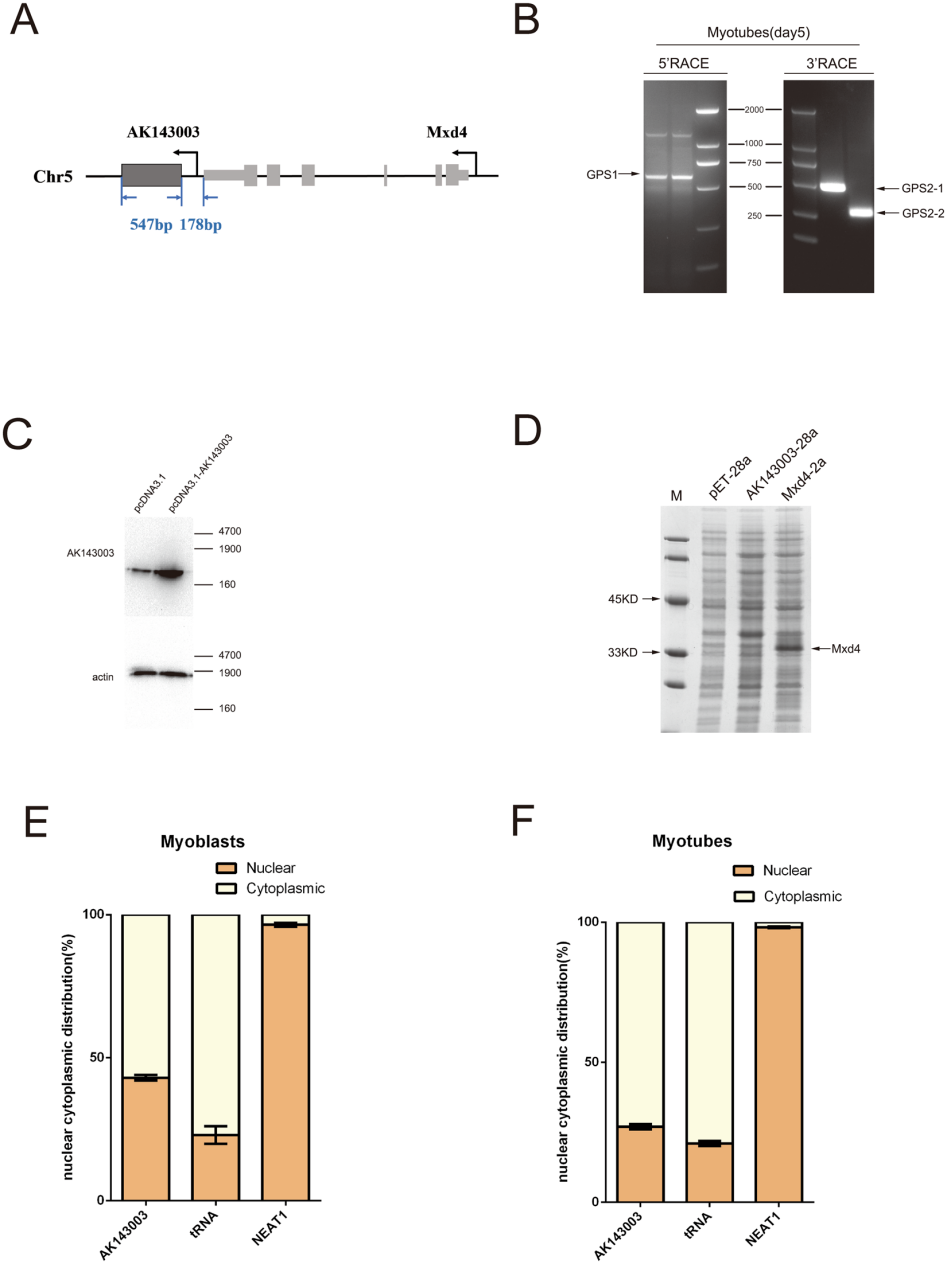
The characteristics of AK143003. (**A**) A sketch about the DNA sequence structure of AK143003. (**B**) Agarose gelelectrophoresis showing the 5′ and 3′ RACE results of AK143003 for 5 days differentiation. (**C**) Northern blot analysis of AK143003, which was extracted from 3 days differentiation of C2C12 cells after transfected pcDNA3.1-AK143003 or control vector. Actin was used as a loading control. (**D**) *In vitro* translation system showing no evidence of protein product with AK143003, while Mxd4 was proved as a coding protein. Actin was the positive control of the experiment. (**E** and **F**) RNA of C2C12 cells were fractionated into nuclei and cytosol fractions. AK143003 subcellular distribution were calculated by qPCR assay data, the graphs showed the expression in proliferative cells (**E**) and differentiated cells of 3 days (**F**), Cytoplasmic control (*tRNA*) and nuclear control (*NEAT1*) were examined in their expected localization.

The microarray chip and tissue expression profiles indicated that AK143003 expression occurred according to the dose of MyoD. Based on the sequence from NCBI, AK143003 contains one exon and is transcribed from mouse chromosome 5, close to a protein-coding gene, *Mxd4* (Fig. 3A). To identify the entire full-length cDNA sequence of AK143003, we performed 5′ and 3′ RACE. The results revealed that AK143003 is a 547-nucleotide transcript that lacks an intron, its start site begins with a guanine, and it has a polyadenylated tail like many lncRNAs (Fig. 3A and B). This result was in close agreement with the database sequence. To further confirm this conclusion, Northern blot analysis revealed that AK143003 was a single transcript (Fig. 3C).

Next, we used two proven algorithms to predict the protein coding potential of AK143003. The Coding Potential Calculator (CPC) tool indicated that lncRNA-AK143003 is probably a non-coding RNA, similar to lncRNA-*HOTAIR* (Supplementary Fig. 3A). In addition, there were no open reading frames, and the Kozak strength in the AK143003 sequence did not match any known proteins in the current proteome databases. Using an *in vitro* translation system, we failed to produce any protein product (Fig. 3D), further supporting its noncoding property. RNA folding analysis revealed the existence of abundant stem-loop structures of AK143003, indicating that it could form a complex tertiary structure to engage in a variety of biological functions (Supplementary Fig. 3B).

To determine the cellular location of AK143003, we partitioned the nuclei and cytosol RNA from C2C12 cells. Real-time PCR analysis showed that the AK143003 transcripts were localised nearly equal amounts in the cytoplasm and nucleus during the proliferative stage, whereas well-known nuclear lncRNA-*NEATI* and cytoplasmic lncRNA-*tRNA* were found to be highly enriched in nuclear and cytoplasmic fractions, respectively (Fig. 3E). Although a previous study reported difficulty in fractionating differentiated cells, we successfully obtained compartmentalised RNAs from differentiation cells at 3 days. It displayed that almost all AK143003 transcripts were enriched in the cytoplasmic extracts, along with *tRNA* (Fig. 3F). These results suggest that AK143003 transferred from the nucleus to the cytoplasm, likely playing a functional role during myocyte differentiation.

### AK143003 is a novel lncRNA for skeletal muscle cell differentiation

**Figure 4:**
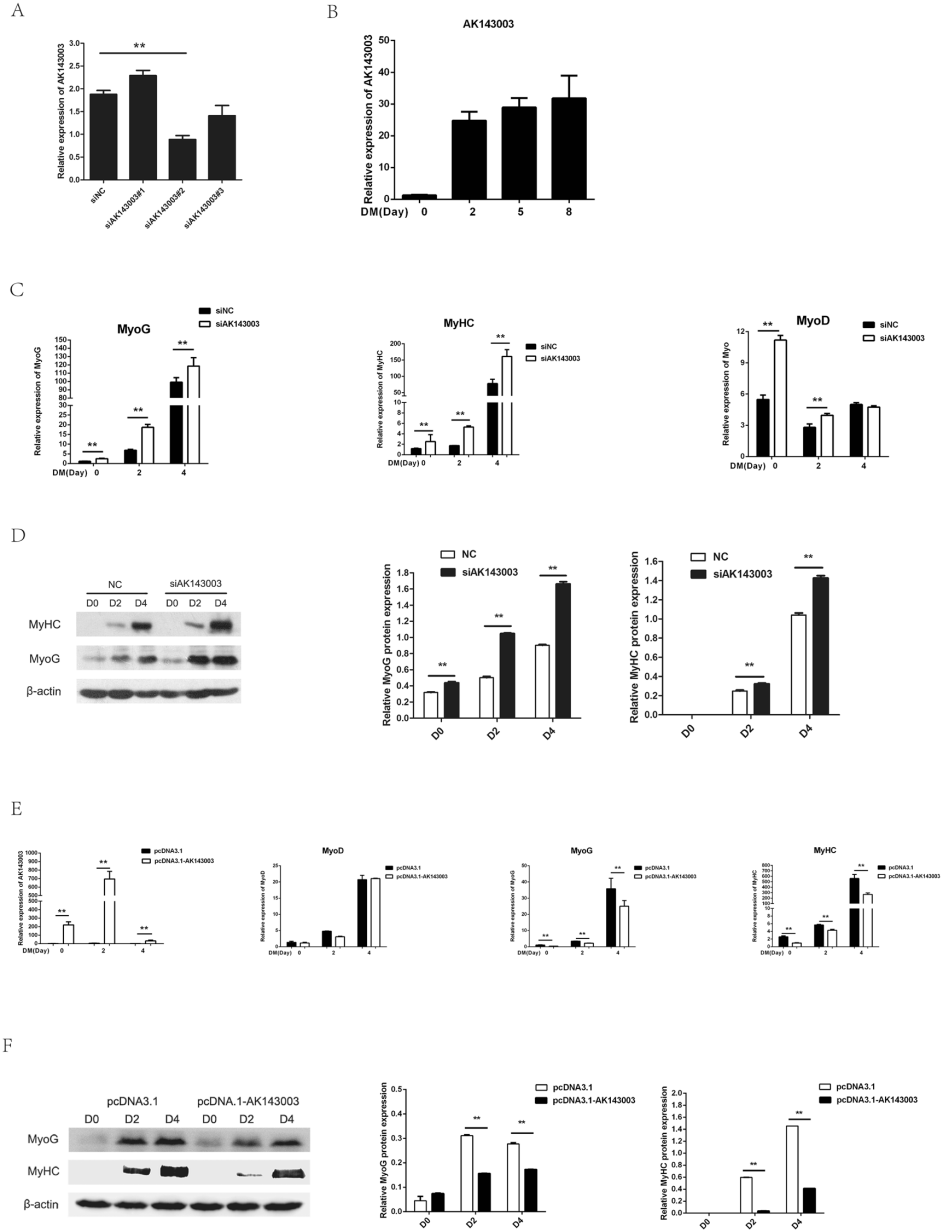
The function of AK143003 during C2C12 myoblasts differentiation. (**A**) Screening assay of siRNA of AK143003 showed the second siRNA have the highest interference efficiency. (**B**,**C**) the result of AK143003 interference during a 4-days differentiation course was showed in (**B**). The myogenic marker gene were upregulated after AK143003 silencing (**C**). (**D**) Knockdown of AK143003 increased the levels of MyoG and MyHC protein during a 4-days differentiation course. (**E**) The AK143003 overexpression results at diverse differentiation time. (**F**) The mRNA levels of skeletal muscle marker genes were decreased in cells from E. (**G**) Western blot result of indicated genes protein after enhancing the expression of AK143003. All the qPCR data were normalized to *β-actin* and presented as mean ± S.D. (n = 3). *p < 0.05, **p < 0.01. The relative expression of protein levels represented by ratio of gray value to β-actin.

The early inductions of AK143003 during cell differentiation have suggested that it might initiate myogenesis and maintain differentiation. To test this hypothesis, we generated three independent small interfering RNA (siRNA) to target different regions of AK143003. Among them, one siRNA showed the highest knockdown efficiency and was used for subsequent experiments (Fig. 4A). The cells were transfected with the siRNA to inhibit AK143003 expression over the course of differentiation (Fig. 4B). *MyoG* and *MyHC* were both increased at the mRNA level. *MyoD* mRNA expression was activated at the early stage of differentiation but not changed in following stages (Fig. 4C). As expected, the protein expression of MyoG and MyHC increased upon AK143003 knockdown (Fig. 4D).

To further verify the results of AK143003 knockdown, we overexpressed the full-length to monitor the marker gene expression. Promoting the expression of AK143003 not changed the mRNA expression of *MyoD*, but led to a delayed differentiation as assessed by MyoG and MyHC mRNA and protein expression (Fig. 4E,F). These results indicated that AK143003 could act as a negative regulatory factor to regulate muscle differentiation.

## Discussion

Recent studies using large-scale high throughput technologies have identified lncRNAs as a group of epigenetic regulators involved in multiple biological functions^35–37^. Most studies about lncRNAs have focused mainly on carcinogenesis and various human cancers, with little known about their role in myogenesis. Trapnell *et al*. reported a lot of unannotated lncRNAs through high-throughput sequencing during C2C12 cell differentiation^38^; later, other researchers used sequential integration and mining of sequencing information to identify potentially functional lncRNAs associated with differentiation^30, 31^. Among them, *lncMyoD* was found to be located proximal to *MyoD* and to promote terminal differentiation by *cis*-acting regulation of MyoD, suggesting the important role of MyoD-related lncRNAs in muscle differentiation. In order to identify and understand the lncRNAs involved in myogenesis, we designed a microarray platform using *MyoD*-inhibited or -overexpressed cells and investigated the role of lncRNAs in affecting epigenetic changes in muscle.

To our knowledge, this is the first report to identify MyoD-mediated lncRNAs in myogenesis. Genomic location analysis revealed that the most differentially regulated lncRNAs originated from intergenic and coding gene exons, and some were located close to muscle-specific genes. Earlier studies on lncRNAs demonstrated they could regulate neighbor genes in *cis* to perform their functions^39, 40^. Nevertheless, whether these genes exert *cis*-acting abilities to impact muscle differentiation requires further analysis.

By performing clustering analysis of differentially co-expressed genes, we found most genes converged into myogenesis or skeletal muscle processes, while others were involved in the activation of cell cycle processes. These results aligned with our expectation because the basic function of MyoD is to induce cell cycle withdrawal to trigger differentiation. For instance, genes in the *actin*, *troponin*, and *MyHC* families were significantly changed after the knockdown of *MyoD*. These genes enriched of GO terms associated to “skeletal muscle contraction”, “contractile fiber”, “myofibril”, and were found to participate in “focal adhesion”, “leukocyte transendothelial migration” and “adrenergic signaling in cardiomyocytes” pathways. Notably, some cell cycle factors including *CCND3*, *CDC25A*, *CDC6*, *CDK2*, and *CDKN1C* were enriched in cell cycle signaling pathway, indicating some transcripts may affect cell differentiation by controlling the cell cycle. Taken together, MyoD-regulated lncRNAs could affect muscle differentiation through multiple pathways.

Recent genetic studies showed intergenic lncRNA may act as tissue-specific molecules compared with coding genes^41^. Therefore, the MyoD-mediated lncRNAs should be enriched in muscle tissue. However, as an important transcription factor, MyoD has the ability to induce various progenitors to convert into myogenic cells^42^. Therefore, it is possible that MyoD-monitored lncRNAs were highly expressed in other non-muscle tissues. The results of tissue-expression profile in 37 selected lncRNAs were consistent with our expectations. From the qPCR analysis, we found nine lncRNAs with higher expression in muscle, of which one (AK141672) was demonstrated a heart-specific expression lncRNA. In addition, AK160312, AK163925, ENSMUST00000154720, ENSMUST00000104935 and MM9LINCRNAEXON11961− were highly expressed in all kinds of muscle tissues. It indicated these lncRNAs could play an important role in muscle development. Interestingly, AK017263, AK031663, and ENSMUST00000150337 also displayed a higher abundance in adipose tissue. Some studies have shown that MyoD could convert adipose tissue to muscle tissue^39, 40, 43^. And brown fat cells were derived from the population of muscle stem cells^43–45^. For adipocyte some researchers found the Myf5 is required for restarting fat metabolism and maintaining energy in thermoregulation^46, 47^. Furthermore, it could effectively inhibit myogenic transformation by decreasing the expression of MyoD^48^. These studies suggest the potential function of the three lncRNAs in mutual transformation between adipocytes and myocytes. Besides that, we also found a group of transcripts (AV570737, UC008MAU.1, ENSMUST00000053965, ENSMUST00000118172, ENSMUST00000118351, ENSMUST00000168516, MM9LINCRNAEXON11909− and AK118572) exhibited predominant expression in testis. As steroid hormone secreted from testes can promotes muscle growth^49^, it is plausible that these genes are linked with muscle hypertrophy induced by androgenic hormone. Mechanistically, similar reasons may account for the highly expression in brain tissue. Finally, numerous lncRNAs, such as *H19* and *NEAT1*, showed ubiquitous expression and were conserved in evolution^50–53^. This expression pattern suggests that they may participate in basic biological processes, including growth, development, and cell multiplication. These genes could be regarded as candidate genes for follow-up study.

In this study, we focused on AK143003, which was shown to be a non-coding RNA. It was negatively regulated by MyoD, and it inhibited muscle marker genes, *MyoG* and *MyHC*. Notably, the dynamic expression of AK143003 influenced MyoD expression only at the early differentiation stage, whereas MyoG and MyHC expression could be regulated during cell differentiation, with peaking enrichment appearing after differentiation for 2 days. Thus, we hypothesize that AK143003 plays a functional role mainly at the early stage of differentiation following *MyoD* induction. In addition, subcellular localisation analysis revealed that AK143003 was enriched in the cytoplasm, regardless of proliferation or differentiation conditions. So we speculate two types of regulation mechanisms: (i) binding to the mRNA with RNA binding proteins (such as Staufen1) to promote or prevent decay of mRNA^54–56^, which could impair myogenesis through regulating c-myc^57^; or (ii) competing endogenous miRNAs as a miRNA sponge to regulate the expression of target genes, such as *lnc-MC* ^58, 59^. Using the probability of interaction by target accessibility (PITA) software, we analyzed a variety of potential binding sites of miRNAs for targeting AK143003 with a highly matched-degree in seed sequences. This analysis included *miR-181*, *miR-24* and *miR-29*, which have been reported to promote differentiation^60–62^. Nevertheless, further evidence is required to support the hypothesis.

This study provided the first comprehensive analysis of whether MyoD could mediate lncRNAs to impact muscle differentiation. We predicted the potential function of differentially expressed lncRNAs by bioinformatics and tissue expression profile analysis. Finally, one novel lncRNA-AK143003 was found to play an important effect in regulating muscle differentiation.

## Materials and Methods

### Animal and tissue preparation

The Kunming mice were treated according to the guidelines of Good Laboratory Practice with nutritional food and sufficient water. Animal feeding and testing processes were conducted based on the National Research Council Guide for the Care and Use of Laboratory Animals, and were approved by the Institutional Animal Care and Use Committee at Huazhong Agricultural University. After 3 months of careful breeding, the mice were sacrificed. Thirteen tissues—including heart, liver, spleen, lung, kidney, brain, tongue, *longissimus dorsi*, leg muscles, fat, stomach, intestine, and testis, were collected and stored in liquid nitrogen, then grounded into powder by mortar and pestle. All samples were transferred to tubes containing TRIzol.

### Plasmid constructs and siRNA synthesis

The *MyoD* eukaryotic expression vector was constructed using cDNA from C2C12 cells in the proliferation period. The CDS sequence of *MyoD* was amplified with MyoD-F/R primers (forward primer, 5′-GGGTACCGCCACCTCCGTGTTTCGACTCACCAG-3′ and reverse primer, 5′-GCTCGAGTCAAAGCACCTGATAAATCG-3′). Then, it was cloned into the *Kpn*I and *Xho*I sites of the pcDNA3.1 vector using T4 DNA Ligase (Takara, Japan).

The AK43003 vector was constructed using cDNA from C2C12 cells that had been differentiated for 5 days. The full-length of AK143003 was obtained using the RACE assay, and it was amplified with the following primers: forward primer, 5′-GCTAGCGGAAGGAAGGAAGGAA-3′ and reverse primer, 5′-GGATCCAAGAAAGGAAGTTGAGAAAGC-3′. Then, it was cloned into the *Nhe*l and *Bam*HI sites of the pcDNA3.1 vector using T4 DNA Ligase. *MyoD* siRNA were synthesised as described previously^63^. The siRNAs were as follows: AK143003-si1 (sense), CCGUUACGUGUACAUCCAATT and AK143003- si1 (antisense), UUGGAUGUACACGUAACGGTT; AK143003-si2 (sense), GCACAAAUACACAUGGACATT and AK143003-si2 (antisense), UGUCCAUGUGUAUUUGUGCTT; AK143003-si3 (sense), ACUGGGUCCUCCAAGCUUUTT and AK143003-si3 (antisense), AAAGCUUGGAGGACCCAGUTT.

### Cell culture and differentiation

The mouse C2C12 cell line was provided by the cell bank of the Chinese Academy of Science and cultured in high-glucose Dulbecco’s Modified Eagle’s medium (DMEM; Hyclone, USA) supplemented with 10% foetal bovine serum (FBS) at 37 °C in 5% CO_2_.

For differentiation, the culture medium was replaced with DMEM containing 2% horse serum.

### Cells transfection

Cells were transfected with siRNA or plasmid using Lipofectamine 2000 (Invitrogen, USA). C2C12 cells were seeded in 6-well plates and cultured until the cell density reached ~60–70%; cells were transfected after 12 h with 4 μg of expression vector or approximately 1.44 µM siRNA oligo (GenePharma, China) in each well. After incubating for ~6 h, the cell media were replaced with culture media containing serum. Transfection efficiency was measured using the GFP vector after 48 h under the same transfection conditions.

### Microarray and computational analyses

This study used Mouse LncRNA Microarray V2.0, which contained 31,423 lncRNAs and 25,376 coding transcripts. The transcripts were collected from various databases including RefSeq, UCSC Knowngenes, Ensembl and other related literature. Total RNA was isolated using TRIzol (Invitrogen, USA) from C2C12 myoblasts that had been differentiated for 2 days with *MyoD* siRNA oligo. The quantity and quality were measured by NanoDrop ND-1000 and the integrity was assessed by standard denaturing agarose gel electrophoresis. After testing interference efficiency, three siNC (siRNA of negative control) samples and three siMyoD samples were selected to chip hybridization. Each sample was purified and transcribed into fluorescent cRNA along the entire length of the transcripts without 3′ bias, utilising a random priming method. The labelled cRNAs were purified using the RNeasy Mini Kit (Qiagen). The concentration and specific activity of the labelled cRNAs (pMol Cy3/μg cRNA) were measured by NanoDrop ND-1000. Hybridisation solution was dispensed into the gasket slide and assembled to the lncRNA expression microarray slide. The slides were incubated for 17 h at 65 °C in an Agilent hybridisation oven. The hybridised arrays were washed, fixed, and scanned using the Agilent DNA Microarray Scanner (product number G2505C).

Raw data were extracted using Agilent Feature Extraction software (v11.0.1.1). Then, quantile normalisation and subsequent data processing were performed using the GeneSpring GX v11.5 software package (Agilent Technologies). Differentially expressed lncRNAs and mRNAs with statistical significance between the two groups were identified through fold-change filtering (≥1.5), unpaired *t*-test (*p* < 0.05), and multiple hypothesis testing (FDR < 0.05). GO and pathway analyses (https://david-d.ncifcrf.gov/) were performed to identify differentially regulated biological processes. GO analysis covered three domains: biological processes, cellular components, and molecular functions. The *p*-value denoted the significance of GO terms enriched in the differential expression of the gene. Pathway analysis was based on KEGG, and the *p*-value denoted the significance of the pathway (cut-off, *p* < 0.05).

### RNA isolation and quantitative real-time PCR (qPCR)

Total RNA was isolated from C2C12 cells and tissues using TRIzol reagent (Invitrogen, USA). The concentration and quality were measured by NanoDrop 2000 and agarose gels electrophoresis. The first strand cDNA was generated with PrimeScript RT reagent kit (Takara, Japan) according to the manufacturer’s instructions. Quantitative real-time PCR (qPCR) was performed using the THUNDERBIRD™ probe qPCR Mix or SYBR^®^Green Real-time PCR Master Mix (Toyobo, Japan) protocol on an Applied Biosystems StepOnePlus Real-Time PCR system. The primers used in the assay were listed in Supplementary Table 3. *β-actin* was used as an endogenous control to normalise the basal level. All experiments were performed in biologic quadruplicate. The relative expression of RNAs was calculated using the Ct (2^−ΔΔCt^) method^64^.

### 5′ and 3′ RACE and full-length lncRNA cloning

5′ and 3′ rapid amplification of cDNA ends (RACE) was performed using the Takara SMARTer RACE cDNA amplification Kit (Clontech) according to the manufacturer’s instructions. RNA was extracted from cells that had been differentiated for 5 days, and the stability was checked to ensure that it was free of contaminants. The gene-specific primers used for PCR were as follows:

5′ RACE (ACCAGGCAGAGCTGTCTGGACTGGCC)

first 3′ RACE (GTGGGTGGCCCCCTTTTCCATGGTGTCA)

second 3′ RACE (AATGGTGGCCCCTAACCTGTGCCTGACT)

The PCR products were separated on 1.5% agarose gels, and the bands were extracted and inserted into the pMD-18T vector. Positive colonies were selected for sequencing. Sequences were aligned with BLAST in the NCBI standard nucleotide BLAST.

### Northern blot analysis

Cells were transfected with pcDNA3.1-AK143003 or vector, and the RNA kit (Takara) was used to isolate total RNA from C2C12 cells that had been differentiated for 3 days. A total of 4.5 µL RNA was combined with 2 µL 10× MOPS buffer, 3.5 µL formaldehyde, and 10 µL deionised formamide in a total volume of 20 µL. Samples were heated at 60 °C for 10 min, cooled on ice for 2 min, added to 3 µL 10× RNA loading buffer, then fractionated on 1.2% formaldehyde-agarose gels at 7.5 V/mL. After transferring to a positively charged nylon membrane and UV cross-linking, the digoxigenin (DIG)-labelled probe was hybridised to the membrane at 60 °C overnight. The single-strand RNA probes are listed as follows: AK143003, 5′-ACCCACTAGATGCTCATCCAAGTCCTGCCCCTCACAGATCCCTC-3′; actin, 5′-CGTCCCAGTTGGTAACAATGCCATGTTCAATGGGGTACTTCAGGGTCAGG-3′.

### *In vitro* translation system

The full-length of AK143003 and *Mxd4* CDS were inserted into the pET28a vector using the restriction enzyme sites *Eco*RI and *Xho*I. After transformation, single clones were transferred to liquid medium, incubated at 37 °C and 200 rpm until the optical density (OD) value reached 0.6–0.8, and then continuously induced with isopropyl-β-D-1-thiogalactopyranoside (0.5 mM) for 2 h. The samples were centrifuged at 12,000 *g* for 1 min. The sediments were re-suspended in 10 mM Tris-HCl (pH 8.0), and the supernatants were subjected to electrophoretic separation.

### Western blot analysis

Proteins from the cell lysates were prepared in 100 mg/mL RIPA lysis buffer containing 1% (v/v) phenylmethylsulfonyl fluoride (Beyotime, China), then centrifuged at 12,000 *g* for 10 min at 4 °C. After that, the lysates were heated at 95 °C for 5 min in 5× sodium dodecyl sulfate (SDS) sample buffer. Identical quantities of proteins were separated by 10% SDS-PAGE gel and transferred onto polyvinylidene fluoride (PVDF) membranes (Millipore, USA), and non-specific binding was blocked with 5% non-fat milk in Tris-buffered saline with Tween 20 for 4 h. The membranes were incubated overnight with primary antibodies at 4 °C, washed three times, and then incubated with the secondary antibodies for 1 h at 37 °C. The antibodies for Western blot analysis were obtained from Santa Cruz Biotechnology, including anti-myogenin (sc-12732; 1:200 dilution), anti-MyoD (sc-760; 1:200 dilution), anti-MyHC (sc-376157; 1:1000 dilution), and anti-β-actin (sc-4777; 1:1000 dilution). Changes in protein levels were normalised to the housekeeping protein β-actin for quantitative Western blot analysis using ImageJ software.

### Nuclear and cytoplasmic RNA fractionation

Cells were prepared for proliferation and differentiation, washed twice with cold PBS, and centrifuged at 500 *g* for 3 min. The liquid precipitation was gently resuspended in 0.2 mL lysis buffer (50 mM Tris-HCl pH 8.0, 140 mM NaCl, 1.5 mM Mgcl_2_, 0.5% IGEPALH CA-630, 1U/μL RNase inhibitor, 1 mM DTT), placed on ice for 5 min, and centrifuged at 500 *g* for 3 min at 4 °C. The supernatant was transferred to a fresh 1.5 mL microcentrifuge tube without RNase and centrifuged at 14,000 rpm for 4 min. The cytoplasmic RNA was dissolved in the supernatant, and the nuclear RNA was mixed in the precipitation. The deposit was washed with 0.2 mL lysis buffer to determine the nucleus viscosity. The nuclei were checked for integrity (absence of ropiness), and all nuclear and cytoplasmic RNA were extracted with 1 mL TRIzol.

### Statistical analysis

The results are presented as the means ± standard error (SE). Statistical analyses of differences between groups were performed using Student’s *t-*test. The *p*-value < 0.05 was considered to indicate significance.

## Electronic supplementary material

supplementary figures

Supplementary Table 1

Supplementary Table 2

Supplementary Table 3
