# Author Correction: Identification of MyoD-Responsive Transcripts Reveals a Novel Long Non-coding RNA (lncRNA-AK143003) that Negatively Regulates Myoblast Differentiation

**DOI:** 10.1038/s41598-023-28986-2

**Published:** 2023-02-02

**Authors:** Yiwen Guo, Jingnan Wang, Mingfei Zhu, Rui Zeng, Zaiyan Xu, Guoliang Li, Bo Zuo

**Affiliations:** 1 Key Laboratory of Swine Genetics and Breeding of Ministry of Agriculture & Key Laboratory of Agriculture Animal Genetics, Breeding and Reproduction of Ministry of Education, College of Animal Science, Huazhong Agricultural University, Wuhan, 430070 Hubei P.R. China; 2 The Cooperative Innovation Center for Sustainable Pig Production, Wuhan, 430070 China; 3 National Key Laboratory of Crop Genetic Improvement, Agricultural Bioinformatics Key Laboratory of Hubei Province, College of Informatics, Huazhong Agricultural University, Wuhan, 430070 Hubei P.R. China

Correction to: *Scientific Reports* 10.1038/s41598-017-03071-7, published online 06 June 2017

The Supplementary Information files that accompany this Article contain errors, where Supplementary Table 1, Supplementary Table 2 and Supplementary Table 3 are not displaying correctly.

The corrected Supplementary Tables 1, 2 and 3 are linked to this correction notice.

## Supplementary Information

Supplementary Table 1.

Supplementary Table 2.

Supplementary Table 3.

