# Supplementary material for: Identification of MyoD-Responsive Transcripts Reveals a Novel Long Non-coding RNA (lncRNA-AK143003) that Negatively Regulates Myoblast Differentiation
Source: Sci Rep. 2017 Jun 6;7:2828. doi: 10.1038/s41598-017-03071-7 (PMC5460278; doi:10.1038/s41598-017-03071-7)

Identification of MyoD-Responsive Transcripts Reveals a Novel Long Non-coding RNA (lncRNA-AK143003) that Negatively Regulates Myoblast Differentiation

Yi-wen Guo [1], Jing-nan Wang [1], Ming-fei Zhu[1], Rui Zeng[1], Zai-yan Xu [1], Guo-liang Li [3], Bo Zuo [1, 2*]

[1] Key Laboratory of Swine Genetics and Breeding of Ministry of Agriculture & Key Laboratory of Agriculture Animal Genetics, Breeding and Reproduction of Ministry of Education, College of Animal Science, Huazhong Agricultural University, Wuhan, 430070 Hubei, P.R. China

[2] The Cooperative Innovation Center for Sustainable Pig Production, Wuhan 430070, China

[3] National Key Laboratory of Crop Genetic Improvement, Agricultural Bioinformatics Key Laboratory of Hubei Province, College of Informatics, Huazhong Agricultural University, Wuhan, 430070 Hubei, P.R. China.

*To whom correspondence may be addressed: Dr. Bo Zuo, Key Laboratory of Swine Genetics and Breeding of Ministry of Agriculture & Key Laboratory of Agriculture Animal Genetics, Breeding and Reproduction of Ministry of Education, College of Animal Science, Huazhong Agricultural University, Wuhan, 430070 Hubei, P.R. China

Telephone: +86 027-87282091 E-mail: zuobo@mail.hzau.edu.cn

Supplemental figure legend

Figure S1 (A)Scatter plot showing the distribution of lncRNAs (left) and mRNAs (right) in transfected negative control oligos and *MyoD* siRNA oligo in C2C12 cells during a 48h differentiation. The values of the X and Y axes are averaged normalized values in each group (log2-scaled). The lncRNAs or mRNAs above the top green line and below the bottom green line are those with a > 1.5 fold change in expression between the two groups. (B)Box plot visualizing the lncRNA (left) and mRNA (right) expression variations. After normalization, the distributions of log2-ratios among all samples are nearly the same. (C)The pie chat of the dysregulated lncRNAs distribution in gene location. (D)Chromosomal distribution of the up- and down-regulated lncRNAs or mRNAs. (E) The top 10 enriched pathways for differential lncRNAs coexpressed mRNAs. The bar plot shows the enrichment score (-log10 P value) for each enrichment pathway.

Figure S2. Expression profiles of 46 selected lncRNAs in 13 tissues. Quantitative RT-PCR results were revealed from 3-month-old mice tissue total RNA. LncRNA were divided into several groups A-E) according to the tissue expression pattern. (A) highly expression of lncRNAs in muscle tissue; (B) highly expression of lncRNAs in testis; (C) highly expression of lncRNAs in brain; (D) highly expression of lncRNAs in liver and kidney; (E) widely expressed lncRNAs. Three individuals were prepared for tissue samples. All the qPCR data were normalized to β-actin and presented as mean ± S.D. (n=3).

Figure S3. (A)The Coding Potential Calculator (CPC) score of AK143003, *MyoD* and *HOTAIR*, *HOTAIR* was known as a non-coding RNAs with negative score, MyoD was known as the coding protein. (B) The two-dimensional structure of AK143003 by RNA fold.

Figure S4. Original gels and blots for the Figures in main text.

Supplementary Table 1

The significantly differentially expressed lncRNAs after *MyoD* knockdown

Supplementary Table 2

The significantly differentially expressed mRNAs after *MyoD* knockdown

Supplementary Table 3

The table of primers used for PCR

Supplementary Figure 1

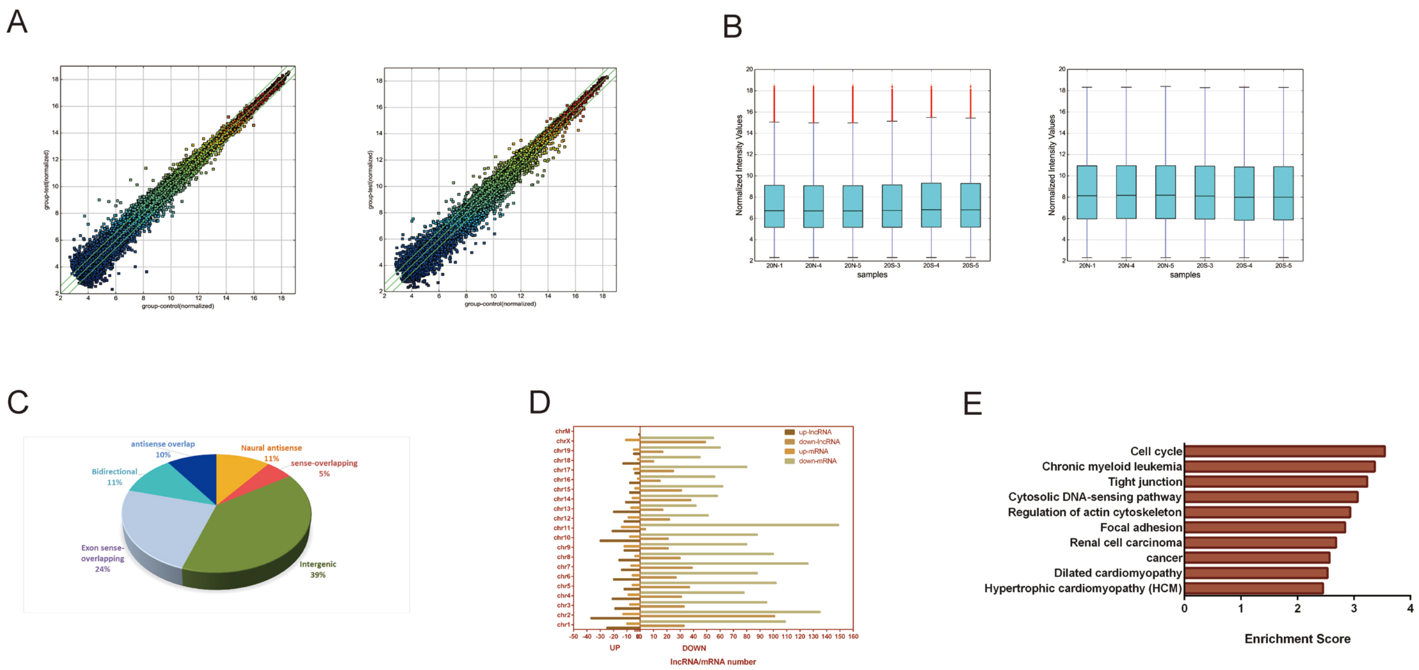

Supplementary Figure 2

A

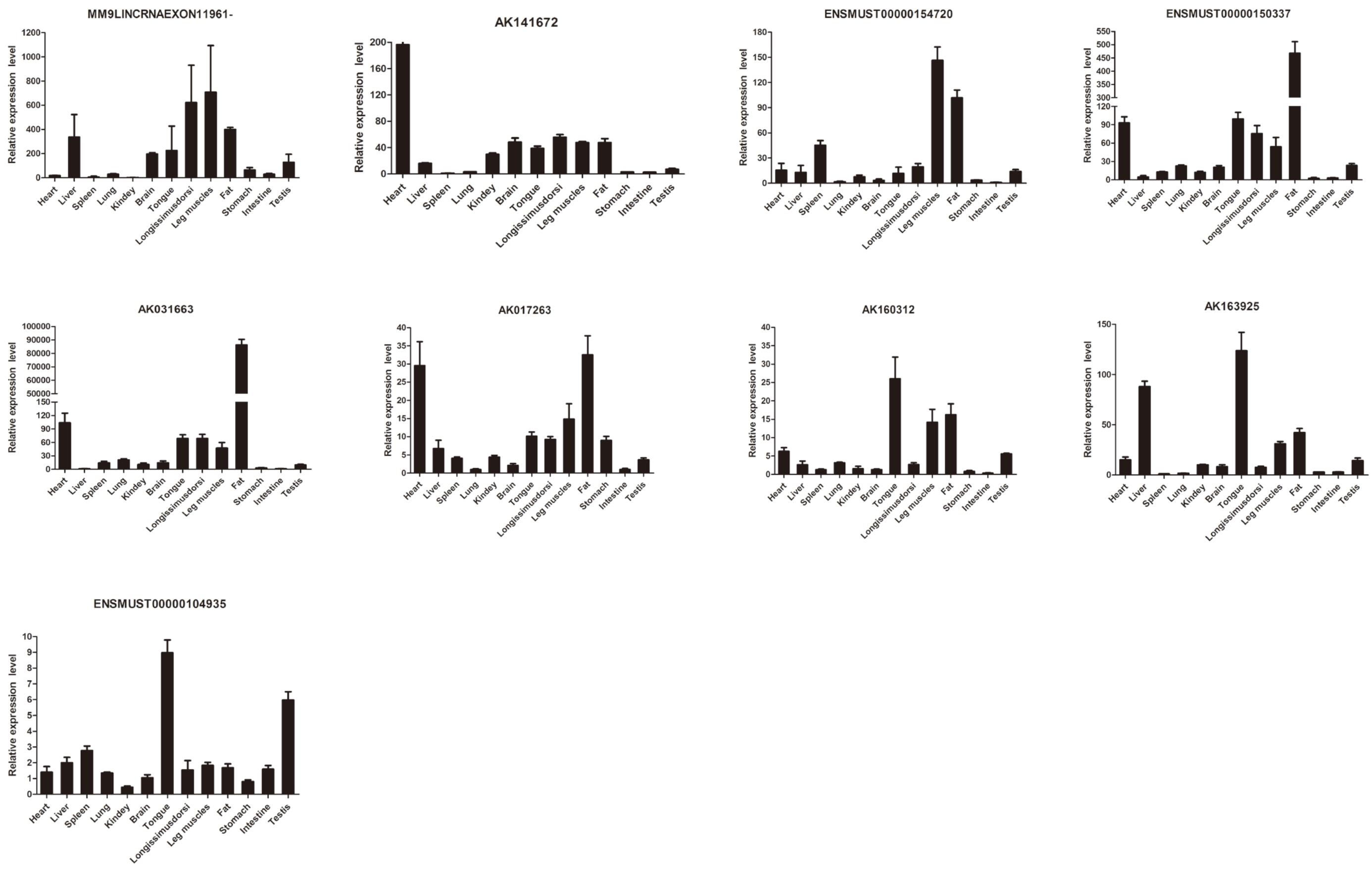

B

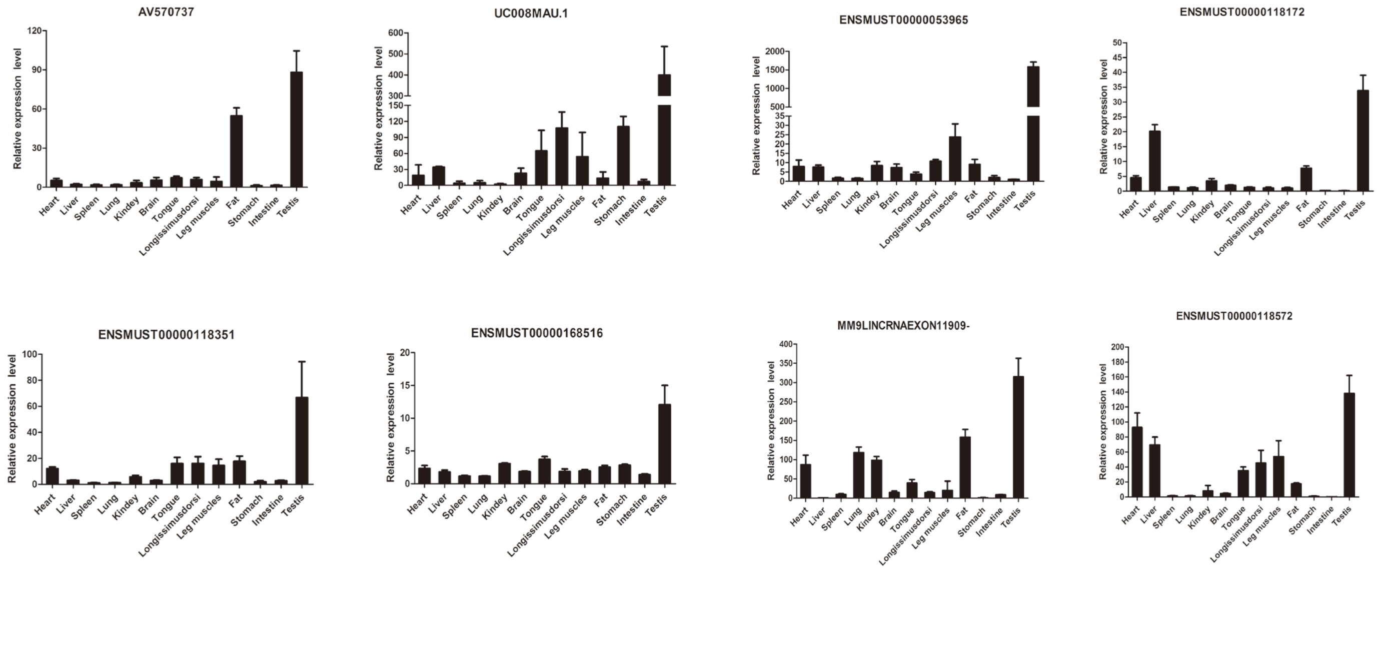

C

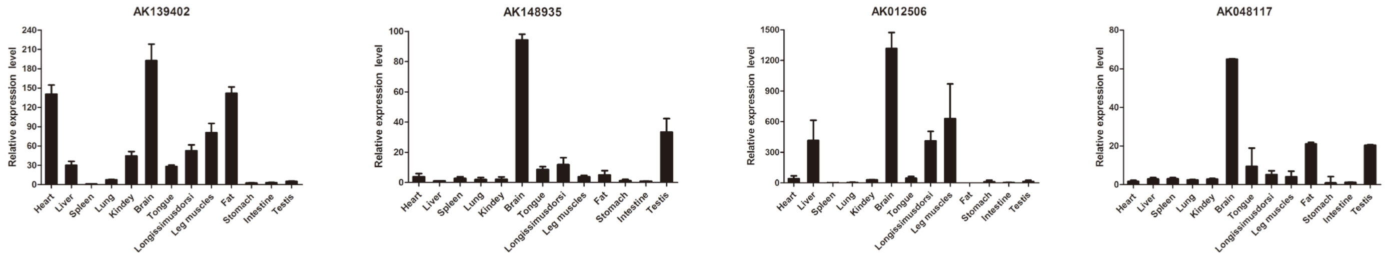

D

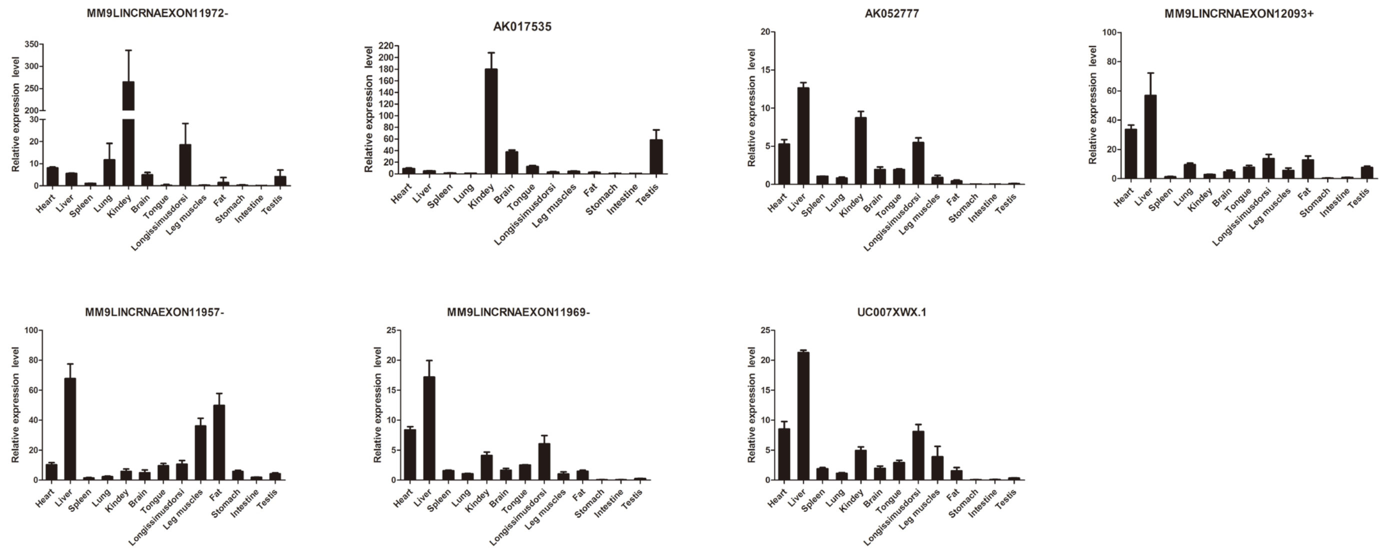
MM9LINCRNAEXON11972-
AK017535
AK052777
MM9LINCRNAEXON12093+
MM9LINCRNAEXON11957-
MM9LINCRNAEXON11969-
UC007XWX.1
Relative expression level
Heart
Liver
Spleen
Lung
Kindey
Brain
Tongue
Longissimusdorsi
Leg muscles
Fat
Stomach
Intestine
Testis

E

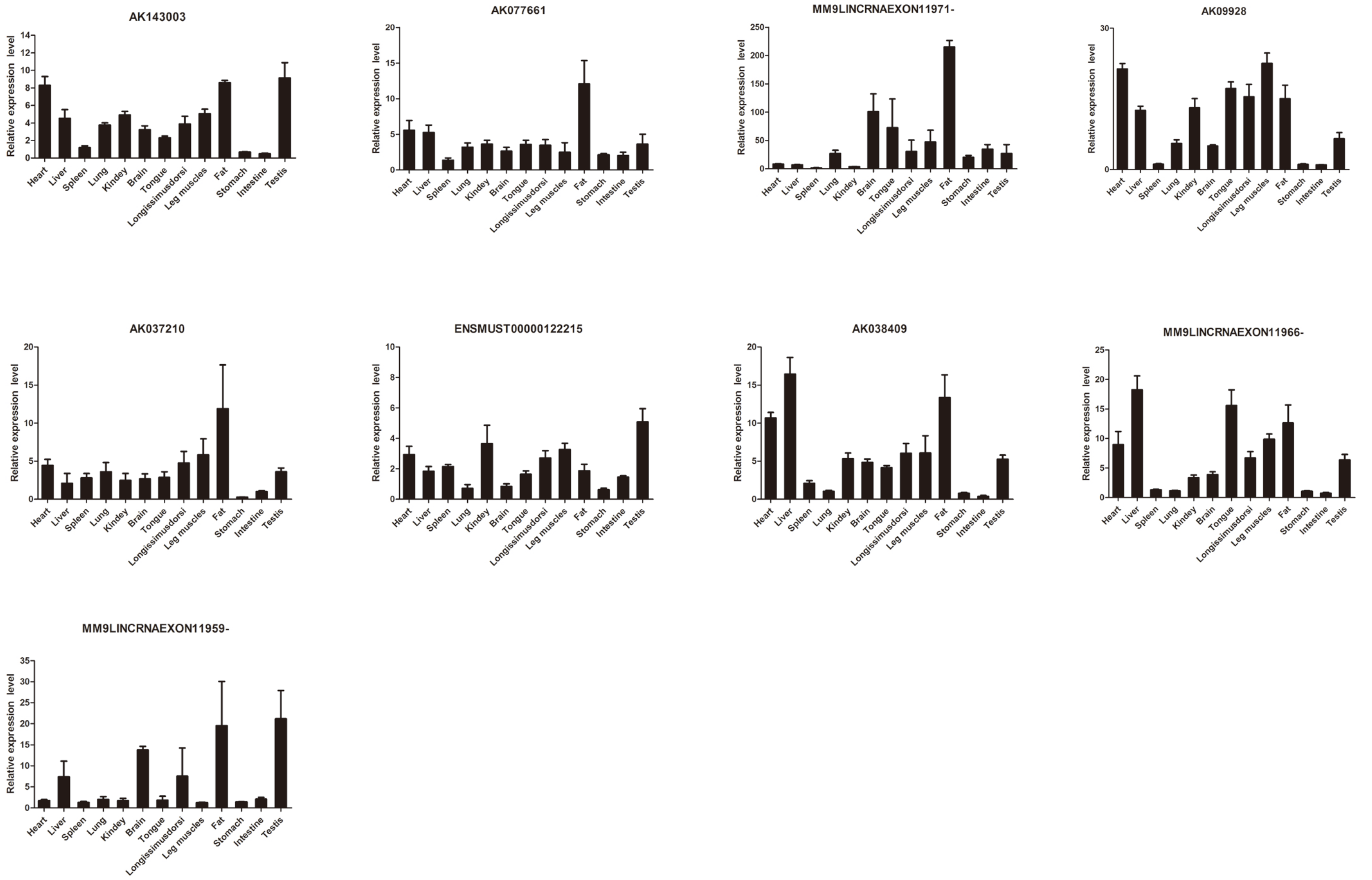
AK143003
AK077661
MM9LINCRNAEXON11971-
AK09928
AK037210
ENSMUST00000122215
AK038409
MM9LINCRNAEXON11966-
MM9LINCRNAEXON11959-
Relative expression level
Heart
Liver
Spleen
Lung
Kindey
Brain
Tongue
Longissimusdorsi
Leg muscles
Fat
Stomach
Intestine
Testis

Supplementary Figure 3

A

ID	C/NC	CODING POTENTIAL
AK143003	noncoding	-0.843319
MyoD	coding	0.691843
HOTAIR	noncoding	-1.18564

B

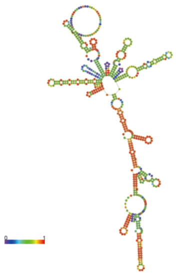

Supplemental Figure S4 Original gels and blots for the Figures in main text

For Fig. 1B

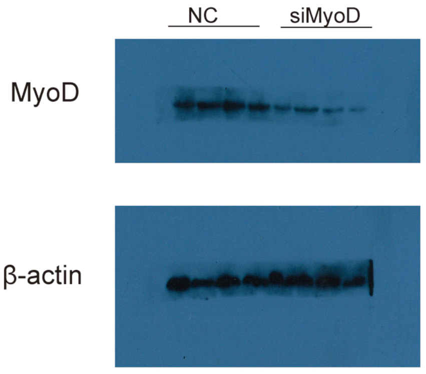

For Fig. 3A

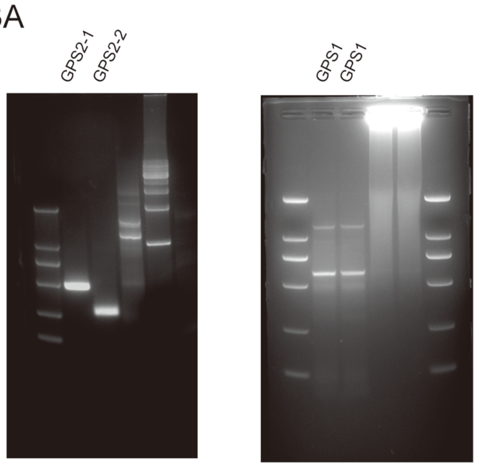

For Fig. 3B

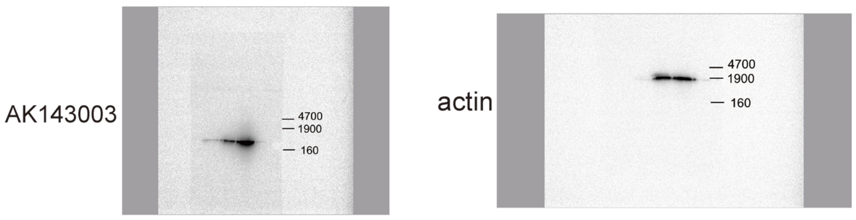

For Fig. 3C

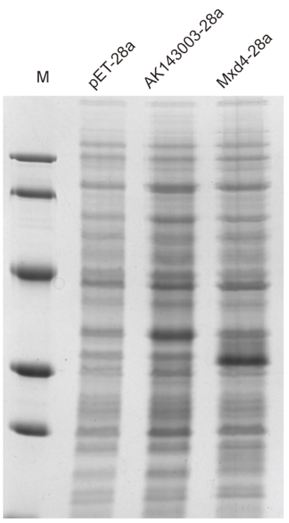

For Fig. 4D

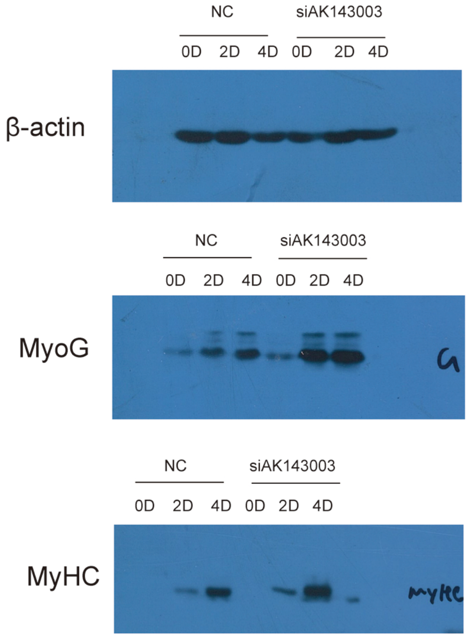

For Fig. 4F

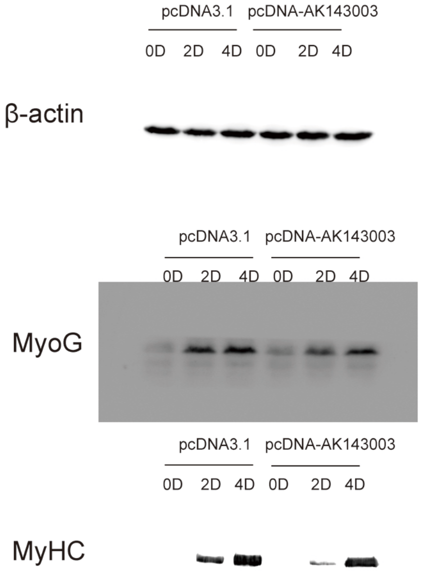

Supplement: Supplementary file 1 — supplementary figures [file 41598_2017_3071_MOESM1_ESM.pdf]
